# Influence of elderly patients' coronary artery lesion severity on cardiac remodeling and left ventricular function

**DOI:** 10.12669/pjms.346.15906

**Published:** 2018

**Authors:** Chao Wang, Xiang Tian, Huixin Cai, Liang Feng

**Affiliations:** 1 *Chao Wang, Baoding First Central Hospital, Baoding 071000, P. R. China*; 2 *Xiang Tian, Baoding First Central Hospital, Baoding 071000, P. R. China*; 3 *Huixin Cai, Baoding First Central Hospital, Baoding 071000, P. R. China*; 4 *Liang Feng, Baoding First Central Hospital, Baoding 071000, P. R. China*

**Keywords:** Elderly, Coronary heart disease, Cardiac remodeling, Systolic function, Diastolic function

## Abstract

**Objective:**

To analyze the correlation between coronary artery lesion and cardiac function change among elderly patients with coronary heart disease (CHD).

**Methods:**

A total of 171 elderly patients with CHD hospitalized from 2009 to 2016 were selected. Their ultrasonic cardiographic and coronary angiographic data were collected, and the correlation between coronary artery lesion and left ventricular remodeling, systolic and diastolic function was analyzed.

**Results:**

Coronary artery lesion among elderly patients with CHD was closely related with left ventricular remodeling and systolic function change, but not significantly correlated with diastolic function change.

**Conclusion:**

Coronary artery lesion severity of elderly patients with CHD was an important reason for left ventricular remodeling and cardiac systolic function change. Early intervention of coronary artery disease is of great significance to protect the heart function.

## INTRODUCTION

Coronary heart disease (CHD) is a serious threat to human health. With the development of drug therapy and coronary revascularization, patients have expanded lifetime, but cardiac insufficiency-induced morbidity keeps increasing.^1^,^2^ Cardiac function change is the main factor influencing CHD patients’ prognosis. Through analyzing the correlation between coronary artery lesion and cardiac function change among elderly patients with CHD, we herein aimed to provide a clinical basis for reasonably proposing treatment regimens and determining the prognosis.

## METHODS

This study was approved by the ethics committee of our hospital, and written consent has been obtained from all patients. A total of 171 elderly patients with CHD hospitalized in Baoding First Central Hospital from 2009 to 2016 were selected, including 122 males and 49 females aged 60-85 years old, of whom 72 had the history of myocardial infarction (MI). All patients had echocardiography and coronary angiography (CAG). Other factors causing LVEF decline, such as valvular heart disease and congenital heart disease, were excluded. Based on ultrasonic Doppler testing, the LVEF values of 62 patients were <50%, indicating decline of left ventricular systolic function.

### Design of questionnaires

Through retrospective investigation, questionnaires were designed to record relevant data of the selected cases.

### Echocardiography examination

The echocardiographic data of patients, including LVEF, LVEDD, LVESD, FS, AOD and LAD, were recorded within one week of hospitalization. LVEDD was used as the main index to evaluate left ventricular remodeling, and LVEF<50% was utilized as the index for decline of left ventricular systolic function. E peak and A peak were detected by a combination of B-type ultrasound, color Doppler and pulse Doppler, and then E/A ratio was calculated as the index for evaluating left ventricular diastolic function.

### CAG examination

CAG was performed by using PHILIPS FD20 digital subtraction angiography. The arteria femoralis or radial artery access was established with the Seldinger technology. The Judkins method was employed for selective CAG, together with multi-position projection. The severity of stenoses was calculated by using QCA, and the degree and range of coronary artery lesions were evaluated based on Gensini scores.

### Statistical analysis

All data were analyzed by SPSS software package, and the quantitative data were expressed as mean ± standard deviation (± S). The means of two groups were compared by the t test. The numerical data were expressed as percentage and subjected to the *χ*^2^ test. Pearson’s correlation analysis was used to analyze the correlation between numerical data. P<0.05 was considered significantly different.

## RESULTS

### Correlation between Gensini score as well as left ventricular structure and function

For elderly patients with CHD, the correlation between Gensini score and LVEDD, LVESD, FS, AOD, LAD, and LVEF had statistical significance (P<0.05). The correlation between Gensini score and E/A ratio was not significant (P>0.05) (Table-I). After excluding MI, the correlation between Gensini score and LVEDD, AOD, LAD, and LVEF had statistical significance (P<0.05). Nevertheless, Gensini score was not significantly correlated with LVESD, FS or E/A ratio (P>0.05) (Table-II).

**Table-I:** Correlation between Gensini score as well as left ventricular structure and function.

Item	Correlation coefficient with Gensini score (r)	P
LVEDD	0.330	0.000
LVESD	0.171	0.032
FS	-0.358	0.000
AOD	0.289	0.000
LAD	0.290	0.000
LVEF	-0.427	0.000
E/A ratio	-0.030	0.738

**Table-II:** Correlation between Gensini score as well as left ventricular structure and function after excluding MI.

Item	Correlation coefficient with Gensini score (r)	P
LVEDD	0.351	0.002
LVESD	0.058	0.640
FS	-0.182	0.156
AOD	0.329	0.005
LAD	0.302	0.010
LVEF	-0.264	0.021
E/A ratio	0.179	0.224

### Comparisons between groups with and without MI history

The groups with and without MI history had significantly different LVEDD, LVESD, FS, AOD, LAD and LVEF (P<0.05). However, their E/A ratios were similar (P>0.05) (Table-III).

**Table-III:** Left ventricular structures and functions of groups with and without MI history

Echo features	Group with MI history	Group without MI history	F	P

	n (X̄±S)	n (X̄±S)		
LVEDD	75 (10.734±16.468)	93 (5.377±5.892)	8.501	0.004
LVESD	68 (4.814±5.436)	90 (2.824±0.740)	11.803	0.001
FS	62 (26.85±8.895)	90 (37.91±8.426)	60.391	0.000
E/A ratio	48 (0.801±0.334)	81 (0.804±0.290)	0.003	0.960
LVEF	76 (46.39±12.976)	94 (66.10±11.960)	105.730	0.000
AOD	72 (5.97±9.025)	89 (3.65±4.963)	4.305	0.040
LAD	72 (7.513±11.169)	89 (4.276±5.638)	5.688	0.018

### Comparisons between groups with and without LVEF decline

The groups with and without LVEF decline had significantly different LVEDD, LVESD, FS, AOD and LAD (P<0.05) (Table-IV). Involvement of interior descending branch, involvement of right coronary artery, multi-branch lesions and entirely occlusive coronary artery differed significantly between the two groups (P<0.05). Nevertheless, the two groups had similar involvement of left main coronary artery and involvement of circumflex artery (P>0.05) (Table-V).

**Table-IV:** Left ventricular structures and functions of groups with and without LVEF decline

Echo features	LVEF <50%	LVEF >50%	F	P

	n (X̄±S)	n (X̄±S)		
LVEDD	60 (11.484±17.131)	108 (5.705±7.395)	9.299	0.003
LVESD	53 (5.356±6.050)	105 (2.835±0.727)	17.844	0.000
FS	49 (23.10±6.521)	103 (38.30±7.618)	144.549	0.000
E/A ratio	36 (0.800±0.354)	93 (0.803±0.287)	0.003	0.956
AOD	60 (6.65±9.661)	101 (3.52±4.787)	7.484	0.007
LAD	60 (8.553±12.332)	101 (4.043±4.829)	10.771	0.001

**Table-V:** Coronary artery lesions of groups with and without LVEF decline.

CAG result	LVEF <50%	LVEF ≥50%	χ^2^	P

	Case No. (percentage)	Case No. (percentage)		
Involvement of left vertical branch	8 (13.56%)	10 (9.17%)	0.769	0.380
Involvement of interior descending branch	59 (96.72%)	88 (80.73%)	8.545	0.003
Involvement of circumflex artery	46 (75.41%)	68 (64.15%)	2.265	0.132
Involvement of right coronary artery	52 (85.25%)	69 (63.89%)	8.744	0.003
Multi-branch lesion	56 (91.80%)	75 (69.44%)	11.181	0.001
Diffuse lesion	40 (65.57%)	40 (36.70%)	13.091	0.000
Entirely occlusive coronary artery	34 (55.74%)	36 (33.03%)	8.328	0.004

## DISCUSSION

Coronary artery lesion may influence cardiac function through severe acute ischemia or long-term chronic ischemia. In order to reduce the outcome of late cardiac dysfunction, doctors have been exploring factors such as negative ventricular remodeling, left ventricular contraction, and diastolic dysfunction. Among these factors, the severity of coronary artery disease is related to ventricular remodeling and left ventricular function.^3^,^4^ In elderly patients with CHD, the left atrium causes constant progression of left ventricle and aorta remodeling and progressive decline of left ventricular systolic function. Cardiac remodeling is the basic mechanism of cardiac failure development, manifested as cardiac chamber expansion and geometrical shape change on echocardiography. Packer et al. reported that the morphological change of ventricular remodeling was earlier than functional change of abnormal hemodynamics.^5^,^6^ Left ventricle remodeling can aggravate the left ventricular systolic dysfunction. CHD patients’ ventricular systolic function in resting condition is of great significance to long-term prognosis. Besides, LVEF decline means increase of death rate.^7^,^8^ LVEF decline in older patients with coronary artery disease is more serious, the vessel lesion and complex lesions were higher than normal heart function group.^9^ In addition, the elderly patients with CHD whose LVEF declined had more serious coronary artery lesion, with more vascular and complex lesions than those of the group with normal cardiac function. The anterior descending branch and right coronary artery lesion was more likely to cause the decline of cardiac systolic function. The similar left main coronary artery lesions of the two groups may be attributed to the small case number. The circumflex artery contributed to 22.3% of ventricular and myocardial ischemia, which was lower than those of anterior descending branch (41.5%) and right coronary artery (36.2%). The ramus communicans connected three blood vessels, which may be responsible for the similar circumflex artery lesions between the two groups.

MI may cause irreversible decrease and pathological hypertrophy of myocardial cells in non-ischemic region, then inducing left ventricular remodeling. In this study, the cardiac chamber expansion and systolic function decline of groups with and without MI history were significantly different. For patients with the same diffuse coronary artery disease, many studies have also confirmed that besides myocardial infarction as a risk factor for left ventricular dysfunction, some traditional risk factors for coronary heart disease, such as family history of coronary heart disease, age, smoking, PCI history, stroke history, history of peripheral vascular disease, chronic complete closure, were also confirmed not associated with left ventricular dysfunction, which may be related to the establishment of collateral circulation, ischemic preconditioning, and ischemic postconditioning.^10^-^13^

For the elderly patients with CHD excluding MI history, there was no partial loss of myocardial cell mass caused by abrupt closure of the coronary artery. The coronary artery lesion degree indicated by Gensini score was significantly correlated with left ventricular remodeling or ventricular systolic function change. Probably, chronic myocardial ischemia caused by arterial constriction was responsible for the cardiac function change, being related with the coronary artery lesion severity. Studies found that for patients with angina pectoris and coronary heart disease, myocardial ischemia degree and cardiac function condition predominantly determined the prognosis.^14^,^15^ Relevant vascular revascularization may be conducive to improving the prognosis.^16^

Large proportions of elderly patients with congestive heart failure but normal ejection fraction mainly suffer from diastolic dysfunction.^17^,^18^ E/A ratio is the mostly used parameter for evaluating diastolic function. Long-term coronary disease, obesity and hypertension can cause myocardial cell ischemia, anoxia, repetitive reperfusion injury and myocardial matrix change, thereby influencing passive accommodation performance and undermining diastolic function.^19^ For patients with early coronary disease, diastolic dysfunction was a more sensitive index than systolic function change.^20^,^21^ We herein found that the coronary artery lesion of elderly patients with CHD did not affect their diastolic function. As systolic function evidently declined, E/A ratios of the two groups remained similar. Furthermore, E/A ratio and LVEF were not significantly correlated, suggesting that the occurrence of diastolic incompetence was unparalleled with systolic function decline. Hence, the causes for diastolic function change among elderly patients with CHD still need in-depth studies.

In conclusion, the coronary artery lesion severity of elderly patients with CHD was an important reason for left ventricular remodeling and cardiac systolic function change. Cardiac diastolic function change was not significantly correlated with coronary artery lesion. It is important in clinical work to improve the blood flow of myocardial tissue and prevent ventricular remodeling to prevent heart failure. At the same time, the early intervention of coronary artery disease is also of great significance to protect the heart function.

### Authors’ contributions

**CW** designed this study and revised the manuscript.

**XT, HC & LF** performed this study and prepared the manuscript.
